# Parisian Medical Intelligence

**Published:** 1860-07

**Authors:** 


### SELECTIONS.

### PARISIAN MEDICAL INTELLIGENCE.

(FROM THE CORRESPONDENCE OF "LONDON LANCET.")

The proceedings of the Academy of Medicine were commenced on May 29th by M. Depaul's termination of the interesting paper, begun at the previous meeting, upon Occlusion of the Os Uteri during Pregnancy necessitating the Use of the Bistoury in order to effect Delivery. A striking feature present in all the three cases detailed was the completeness of the obliteration of the uterine outlet, and also the solidity of the cohesion between the parts. Neither could the smallest trace of the os be detected by the finger, nor did the most powerful uterine efforts, though continued for many consecutive hours, in any way tend to re-establish the normal state of things.

After the reading of this communication, M. Velpeau rose; but as previous notice had been given by M. Blache, physician to the Hospital des Enfans, of an intention to bring forward a claim of priority in favor of Delau over M. Bize, of Montelemart, in the administration of Perchloride of iron in purpura hæmorrhagica, that surgeon waived his right in M. Blache's favor. This latter gentleman stated that several years ago, at the suggestion of M. Delau, he undertook a series of experiments with this remedy, all more or less confirming the successful results detailed in M. Bize's essay. The statement called forth a reply from the reporter of the committee, M. Devergie, in which he maintained that a full investigation of the question of priority had been instituted, and that though M. Delau seems to have been the first to turn his attention to the subject, yet as M. Bize had been the first to *publish* his experiment, the palm must remain in his possession—on the principle, I suppose, of "*Palman qui* (*non*) *meruit ferat.*"

Much time having been fritted away in this tiresome and bootless discussion about priority, it was late when M. Trousseau rose to reply to some of the charges made in M. Devergie's report on the influence of the perchloride of iron, against the partizans of the vitalist or dynamist theory of the mode of action of iron on the economy. The learned professor said that the reporter had treated two questions in his critique: one, the action of the drug; the other, a question, and a most important one, of general therapeutics. As regarded the action of the drug in purpura hæmorrhagica, he did not consider that the cases were sufficient to prove its curative power, and he believed that medical men would be disappointed if they adopted its use and relied upon speedy and certain results. He extolled its merits as a topical hæmostatic, but said that the theory of the *chemical* action of the perchloride was absurd; that its administration in hæmorrhage from the lungs or womb was beyond measure ridiculous; and, in a clever bantering way, followed the course of a supposed dose through the system, from its administration from the mouth to its ultimate distribution, or rather diffusion, amongst all the capillaries of the body, and dwelt upon the improbability of its having reserved the employment of its styptic power until its arrival at the required destination. The sedative effects noted by the author of the paper, M. Trousseau attributed to the cessation of reaction from natural causes, foreign to the action of the remedy administered, and said that he did not believe that any drug possessed both a styptic and at the same time a sedative power. Diverging now into the general question of the mode of action of ferruginous preparations on the system, the professor argued with much power against the views of the chemical theorists, who maintain that iron, when taken, is absorbed directly into the blood and there precipitated as an oxide, and that it restores to that fluid the amount of material necessary for the reconstitution of its globules. By citing the experiments of M. Reveil, who found that in chlorosis the administration of iron, although followed by rapid increase in the quantity of blood-globules, produced no increase in the amount of that mineral in this fluid, M. Trousseau introduced his own theory concerning its therapeutical action,—namely, that of excitation. In chlorosis, where the effects of the iron are best seen, there is no lack of it in the system, but as the globules are few, it is condensed, not diminished; and the special property of the ferruginous compounds is to produce distribution by stimulating the system, and exciting the reformation of the globular ingredient of the blood.

Here the meeting was adjourned to the 5th June.

I was at the Hopital de la Charite this morning (June 4th), and I find that M. Piorry has authorized a medical gentleman, of the name of Guirette, from Lyons, to institute in his wards a series of experiments in order to test the value of a new plan proposed by the said Dr. Guirette, *for the radical cure of phthisis pulmonalis*, during its third or suppurative stage.

Such a proposition, at first sight, is unpromising, and especially at the Charite, whose wards are still echoing with the cruel humbugs and deceptive professions of the Docteur Noir; nevertheless I am disposed to think M. Piorry justified in giving the present scheme a fair trial. The method of treatment consists in the establishment of a fistulous opening through the integuments of the thorax and the pleura into the lung at its diseased part, and in the free admission of atmospheric air into the cavity of the abscess, which at the same time discharges its contents externally. Dr. Guirette was led to believe in the feasibility of such a plan by pure accident. Having at the Hospital at Lyons applied an issue to the chest of a phthisical patient, over the site of a cavity, and having inserted a pea in order to keep up the counter-irritation, this practitioner found that the foreign body had worked its way into the lung, and caused the pus contained in the abscess, which was a very superficial one, to escape at the artificial opening. The result so far from being fatal to the patient, was so beneficial as to lead to complete recovery. He left the hospital apparently cured, and emigrated to Rio Janeiro.

Since the occurrence of this case, Dr. Guirette has applied the penetrating cautery in three instances, and each time with the best success. Encouraged by this experience, he has come to Paris to submit his plan to public criticism; and although he has met with many rebuffs, is now enabled, by the kindness of M. Piorry, to plead his own cause, or rather that of his new system of treatment of this most incurable complaint. Operations have been already commenced; and a lad aged about eighteen years, with all the symptoms of an abscess under the left clavicle, has been handed over to Dr. Guirette, who applied on the third inst., a cautery of Vienna paste in the third intercostal space, towards the axilla. This, when the eschar falls, will be replaced by a pea attached to a thread of silk. I shall report progress in my next.

The following are a few of the practical truths insisted on by M. Desmarres, in his clinical remarks on the results of his experience in ophthalmic medicine, and are worth mentioning:

1. When you see in an obstinate case of conjunctivitis, an elongated clot or streak of mucous adhering to the surface of the eye or lid, be sure that the irritation is due to an eyelash growing out of its natural direction.

2. In a case of monocular palpebral conjunctivitis, the disturbance of the circulation is very often due to an obstruction of some portion of the lachrymal apparatus. Always assure yourself, in these affections, of the permeability of the duct, by means of an injection with Anel's syringe.

3. Never apply nitrate of silver to a recently-prolapsed iris; it often causes the most violent inflammation.

4. After applying nitrate of silver to an eye, whether in solid or liquid form, neutralize the excess by means of a solution of common salt, which forms an insoluble chloride.

5. Never operate for cataract without first seeing if the phosphenes exist, or if there be sugar in the urine. In the first case, the operation is useless; in the last, most dangerous, for the corneal flap is nearly sure to slough.

6. If a patient with dim sight complains of an iridescent halo round the candle, you may prognosticate a glaucoma.—*London Lancet.*

